# Antimicrobial Effects of the Folk Remedy “Amish Amoxicillin” Against *Staphylococcus aureus*

**DOI:** 10.17912/micropub.biology.001753

**Published:** 2025-08-11

**Authors:** Mary M. Decker, Emily G. Ramcharan, Avery T. Stephens, Kevin M. Drace

**Affiliations:** 1 Biological and Environmental Sciences, Samford University, Birmingham, Alabama, United States

## Abstract

The popularity of natural remedies, often amplified by social media, has led many to turn to home treatments like “Amish Amoxicillin” (AA) in place of traditional medicine. Promoted as a “natural” cure for infectious disease symptoms, AA has gained viral attention despite little scientific validation. This study tested the antimicrobial effects of AA against *Staphylococcus aureus* and evaluated the contribution of its individual ingredients. We found that AA has measurable inhibitory activity *in vitro* , primarily due to garlic and vinegar. However, it lacks evidence to support its use over conventional treatments.

**Figure 1. Antimicrobial activity of Amish Amoxicillin (AA) against Staphylococcus aureus .**
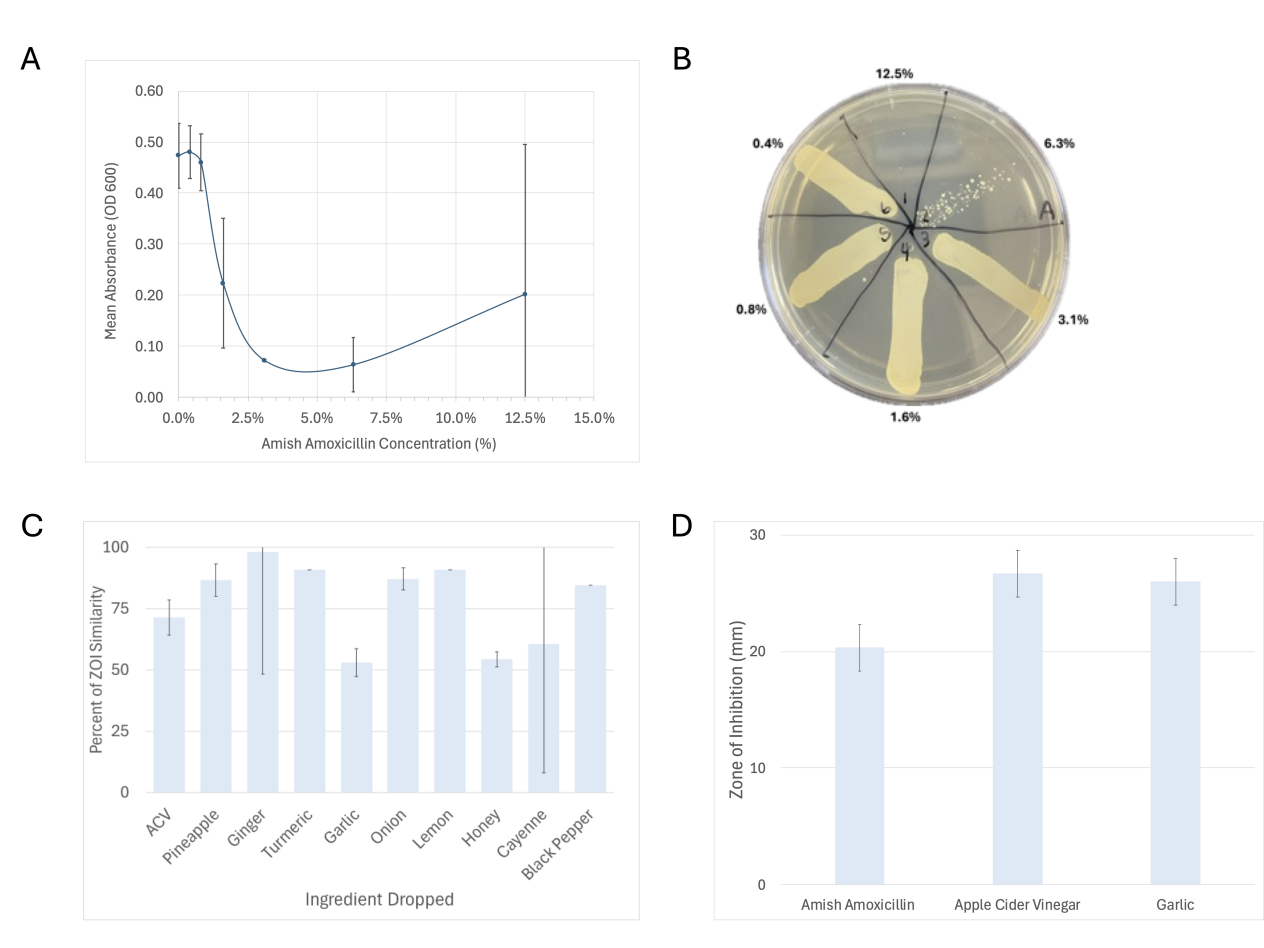
A. Bacterial growth inhibition demonstrated a dose-dependent pattern, with partial inhibition observed above 1.6%. B. Bactericidal activity was tested by subculturing samples from A onto tryptic soy agar plates. No growth was observed at concentrations of 12.5%, indicating complete loss of viability. C. Zones of inhibition measured from well diffusion assays comparing full AA to mixtures with individual ingredients removed shows that the exclusion of garlic, vinegar, or honey reduced antimicrobial activity. D. Zones of inhibition for individual ingredients tested separately with apple cider vinegar and garlic producing measurable inhibition. Error bars represent the standard deviation from the mean.

## Description

Home remedies, often promoted on social media, can gain viral traction as natural alternatives to conventional medicine despite a lack of evidence (Ernst, 2000). One such remedy, commonly referred to as “Amish Amoxicillin” (AA), is a homemade mixture of aromatic vegetables, acidic fruits, and spices blended in apple cider vinegar. Despite claims of its effectiveness, there is little to no scientific research evaluating its antimicrobial potential. Given the risks of replacing clinically tested treatments with unverified alternatives, AA was evaluated to determine whether it exhibits antimicrobial activity and to identify which ingredients, if any, may contribute to that effect.

The antimicrobial properties of AA were tested against *Staphylococcus aureus* , a clinically relevant Gram-positive bacterium often associated with skin and wound infections (Tong, 2015). At concentrations above 1.6% AA inhibited bacterial growth ( Figure 1A ). An increase in absorbance observed at the highest concentrations is likely due to the turbidity of the AA preparation itself rather than bacterial growth, skewing the readings. Bactericidal effects were seen at 6.3% and at 12.5% no growth was observed when samples were transferred to fresh agar plates ( Figure 1B ). These data support that AA is not only inhibitory at sufficient levels but also capable of killing S. aureus under specific conditions.

To assess which ingredients contribute most to AA’s activity, versions of the mixture with individual components removed were tested. When garlic, apple cider vinegar, or honey were excluded, the antimicrobial effect was significantly reduced, suggesting these three ingredients play a critical role ( Figure 1C ). When each ingredient in isolation was tested only apple cider vinegar and garlic resulted in similar zones of inhibition ( Figure 1D ), consistent with prior literature on their antimicrobial properties (Ankri, 1999; Malik, 2024). Honey alone did not inhibit bacterial growth in these experiments, yet its removal from the full mixture diminished the antimicrobial properties. This suggests that honey may enhance the effectiveness through some other means, such as facilitating the diffusion or stability of active compounds (Ahmed, 2018).

These results suggest that while AA does exhibit antimicrobial effects in vitro, its efficacy is largely due to a few well-known antimicrobial ingredients (Malik, 2024). Moreover, the concentrations required to achieve bactericidal outcomes raise practical concerns about its use as a treatment. For example, applying a 12.5% solution topically or ingesting it could pose safety issues due to acidity, irritation, or inconsistency in preparation (Korkmaz, 2000; Johnston, 2008). Unlike standardized pharmaceuticals, home remedies lack dosing guidelines, quality control, and clinical testing, making them unreliable as medical treatments.

These findings do not support the use of AA as a replacement for antibiotics, but they do highlight the value of rigorously testing popular remedies that are widely shared online. Public enthusiasm for natural or homemade treatments should be met with evidence-based evaluation, especially when such remedies are presented as alternatives to antibiotics.

## Methods

**Preparation of Amish Amoxicillin (AA):** The mixture was prepared using a widely circulated recipe found on social media. The following ingredients (weights approximate) were combined in a blender: one medium onion (150 g), one bulb of garlic (30 g), the juice of one lemon (45 mL), ginger root (20 g), turmeric root (20 g), 120 mL of apple cider vinegar, 85 mL of honey, and one teaspoon of cayenne pepper (2 g). Sterile water was added as needed to adjust the consistency, bringing the final blended volume to approximately 400–450 mL. All ingredients were obtained fresh from a local grocery store. After blending until smooth, the mixture was stored in sterile containers at 4°C until use. For ingredient exclusion experiments, the same procedure was followed with one component omitted per condition with the total volume adjusted with sterile water.

**MIC and MLC Determination:** *Staphylococcus aureus* (ATCC 27661) was cultured on tryptic soy agar (TSA) and maintained in tryptic soy broth (TSB) at 37°C. Overnight cultures were diluted to an initial optical density (OD600) of approximately 0.1 before use in antimicrobial assays. To evaluate antimicrobial activity, two-fold serial dilutions of the AA mixture (ranging from 0.8% to 12.5% v/v) were prepared in TSB. *S. aureus* was added to each dilution in triplicate and incubated at 37°C overnight before absorbance readings were measured. To minimize interference from particulate matter, absorbance readings were taken post-incubation without vortexing, when sediment had settled. Minimum lethal concentration (MLC) was determined by streaking each sample onto TSA plates and incubating for an additional 24 hours.

**Agar Well Diffusion Assays:** TSA plates inoculated with *S. aureus* before sterile wells (6 mm) were punched out at regular intervals. Each well was filled with test solutions and plates were incubated at 37°C for 24 hours before zones of inhibition were measured in millimeters. All experiments were performed in triplicate.
